# A Mechanistic Model of *Botrytis cinerea* on Grapevines That Includes Weather, Vine Growth Stage, and the Main Infection Pathways

**DOI:** 10.1371/journal.pone.0140444

**Published:** 2015-10-12

**Authors:** Elisa González-Domínguez, Tito Caffi, Nicola Ciliberti, Vittorio Rossi

**Affiliations:** Department of Sustainable Crop Production, Università Cattolica del Sacro Cuore, Piacenza, Italy; Univeristy of California Davis, UNITED STATES

## Abstract

A mechanistic model for *Botrytis cinerea* on grapevine was developed. The model, which accounts for conidia production on various inoculum sources and for multiple infection pathways, considers two infection periods. During the first period (“inflorescences clearly visible” to “berries groat-sized”), the model calculates: i) infection severity on inflorescences and young clusters caused by conidia (*SEV1*). During the second period (“majority of berries touching” to “berries ripe for harvest”), the model calculates: ii) infection severity of ripening berries by conidia (*SEV2*); and iii) severity of berry-to-berry infection caused by mycelium (*SEV3*). The model was validated in 21 epidemics (vineyard × year combinations) between 2009 and 2014 in Italy and France. A discriminant function analysis (DFA) was used to: i) evaluate the ability of the model to predict mild, intermediate, and severe epidemics; and ii) assess how *SEV1*, *SEV2*, and *SEV3* contribute to epidemics. The model correctly classified the severity of 17 of 21 epidemics. Results from DFA were also used to calculate the daily probabilities that an ongoing epidemic would be mild, intermediate, or severe. *SEV1* was the most influential variable in discriminating between mild and intermediate epidemics, whereas *SEV2* and *SEV3* were relevant for discriminating between intermediate and severe epidemics. The model represents an improvement of previous *B*. *cinerea* models in viticulture and could be useful for making decisions about Botrytis bunch rot control.

## Introduction

*Botrytis cinerea* Pers.: Fr., the anamorph of *Botryotinia fuckeliana* (de Bary) Whetzel, is the causal agent of grey mould, which is a worldwide disease that causes serious losses in more than 200 host species [1,2]. Grey mould (also called Botrytis bunch rot) is one of the most important diseases of grapevines [3].

High genetic variation exists in *B*. *cinerea* populations, and four transposon genotypes (*transposa*, *flipper*-only, *boty*-only, and *vacuma*) have been distinguished [4–11]. The frequency of these transposon genotypes within a *B*. *cinerea* population depends on the growth stage of the grapevine, the organs infected [12], and the vineyard location [9,13].

*B*. *cinerea* infection pathways differ for conidia vs. mycelium; conidia infect inflorescences, young clusters, and ripening berries while mycelium is responsible for berry-to-berry infection [3]. Grape inflorescences are more susceptible at flowering (beginning, full, and end of flowering) than at earlier growth stages or at fruit swelling or berries groat-sized stages [14]. On inflorescences and young grape clusters, infection severity increases with hours of wetness, and the optimal temperature for infection is about 20°C [15–17]. The susceptibility of berries increases from veraison to ripening [18], and at ripening *B*. *cinerea* can infect all berries in a cluster and cause heavy crop losses. Infection incidence in mature grape berries is higher at temperatures between 15 and 25°C than at other temperatures and increases with increasing hours of wetness or high relative humidity [15–17,19,20]. Moreover, disease incidence is higher on wounded than on unwounded berries [19,21–23], especially when wetness is short or humidity is low [17].

Control of *B*. *cinerea* is difficult because: (i) the pathogen can produce large numbers of conidia on multiple inoculum sources (overwintered grape and herb debris, bunch and leaf trash, and rotted berries) [24–29]; (ii) grapevines are susceptible at multiple growth stages [14,30]; (iii) different infection pathways exist [3]; and (iv) infection can occur under a range of environmental conditions, and these conditions differ depending on the infection pathway [14,17,30]. This complexity has caused growers to rely heavily on routine application of fungicides at four specific grape growth stages: A, end of flowering (growth stage 69 of Lorenz et al. [31]; B, pre-bunch closure (growth stage 77); C, veraison (growth stage 83); and D, before harvest (before growth stage 89) [2,20,32–34]. This fungicide scheduling may result in unnecessary sprays [35,36], which is unacceptable because of environmental and public health considerations [37,38] and because of the increased risk of fungicide resistance [39]. There is therefore the need to improve *B*. *cinerea* control in vineyards by applying fungicides only when necessary and by following suitable anti-resistance strategies [39–41].

To reduce the risk of unnecessary sprays, different weather-based methods have been developed. The “15–15 empirical rule” [32,42] and the French EPI-Botrytis model [43] were in some cases associated with a reduction in the number of fungicide applications, but experiments carried out in different countries and seasons were inconsistent [34,43–46]. Other weather-based models have been developed by Broome et al. [20], Nair and Allen [15], and Rodríguez-Rajo et al. [47], but these models lack of robust field validation. An expert system was also developed in Australia, which estimates multiple infection risks based on the available knowledge about *B*. *cinerea* biology, economic thresholds, and fungicides [48]. The expert system was validated in 1990 to 1994 but it was unable to improve the grower’s practice [49].

To date, none of the above models is extensively used by viticulturists. One reason could be the failure of these methods to account for the complexity of *B*. *cinerea* epidemiology, as briefly explained in the previous paragraph (i.e., high sporulation potential, susceptibility of the different grape growth stages, multiple infection pathways, infection occurring under a wide range of environmental conditions, and interactions between environmental conditions and infection pathway). It follows that a method for predicting infection risk by *B*. *cinerea* is still needed.

A new model predicting risk of grapevine infection by *B*. *cinerea* was developed in this research. This model is mechanistic and attempts to account for the full complexity of the *B*. *cinerea* life cycle and of Botrytis bunch rot epidemiology. The model was developed using the results of recent publications that investigated the effect of environmental conditions on the biology and epidemiology of *B*. *cinerea* isolates belonging to different transposon genotypes [14,17,30]. The model was validated against 21 independent Botrytis bunch rot epidemics in Italy and France.

## Model development

### Life cycle of *Botrytis cinerea*

This section briefly describes the key elements of the *B*. *cinerea* life cycle used in model development, with emphasis on inoculum sources, production of conidia, and the main infection pathways described by Elmer and Michailides [3].

*B*. *cinerea* is an inhabitant of vineyards and grows and sporulates under a wide range of environmental conditions [35]. The fungus saprophytically colonizes various organic substrates including grape debris (trash) and weeds covering the soil [3,35,36], and infects both grape and alternative hosts [3].

*B*. *cinerea* mainly survives in grape debris (tendrils, mummified berries, dead leaves, and canes) as over-wintering mycelium [25–28,50] and sclerotia [3,36,51–53]. Abundant conidia are produced on these inoculum sources [3,28,53] under a range of environmental conditions [30]. Mature conidia become airborne with a circadian periodicity (spore concentrations in the air are highest at about midday), which is positively correlated with changes in temperature and wind velocity and negatively correlated with changes in relative humidity and the presence of dew [54]. Airborne conidia can easily travel from field to field [52]. Conidia have been considered the most important form of *B*. *cinerea* inoculum [3,35,47].

Conidia cause infection of inflorescences (at any growth stage) and young berries [3,14,35] through infection path I (conidial infection of the style and ovules), IIa (conidial infection of the stamens or petals), and IIb (fruit infection via the fruit pedicel) [3]. These infections cause either inflorescence blight or they develop into latent infections of berries [29,55–59]. After veraison, latent infections become visible as rotted berries [56,58,60,61].

During the flowering stage, the pathogen also colonizes bunch trash (aborted flowers and fruitless calyptras and stamens), which are retained within the developing bunches (path III: conidial infection and extensive colonisation of floral debris in grape bunches; Elmer and Michailides [3]). *B*. *cinerea* over-summers as saprophytic mycelium on bunch trash, which has been considered a major source of inoculum within developing bunches [29]. Under favourable conditions, the mycelium-colonized bunch trash produces conidia, resulting in inoculation of the external surfaces of the ripening berries (path IV: conidial accumulation within the developing bunch; Elmer and Michailides [3]).

After veraison, a classical pre-harvest polycyclic epidemic can develop under favourable weather conditions; rot develops, and a new crop of conidia are dispersed to new infection sites (pathway V: conidial infection of ripening fruit; Elmer and Michailides [3]). In addition to conidial infection, ripening berries can be infected through contact with the aerial mycelium produced on adjacent infected berries (berry-to-berry infection) [17,18,43,62,63]. Thus, pathway Vb (berry-to-berry infection), which was not described by Elmer and Michailides [3], is included in the new model, and the infection of ripening berries by conidia (former pathway V) is renamed as pathway Va.

Because the life cycle of *B*. *cinerea* includes multiple sources of inoculum, conidia are generally present in the vineyard but at different densities [3,64,65].

Grape is susceptible to *B*. *cinerea* infection during two main periods: during the early developmental stages [66] and from veraison until ripening. In the latter period, susceptibility progressively increases, and this increase has been associated with structural and biochemical changes during berry maturation [18,22,67,68].

### Modeling approach

**Fig 1:**
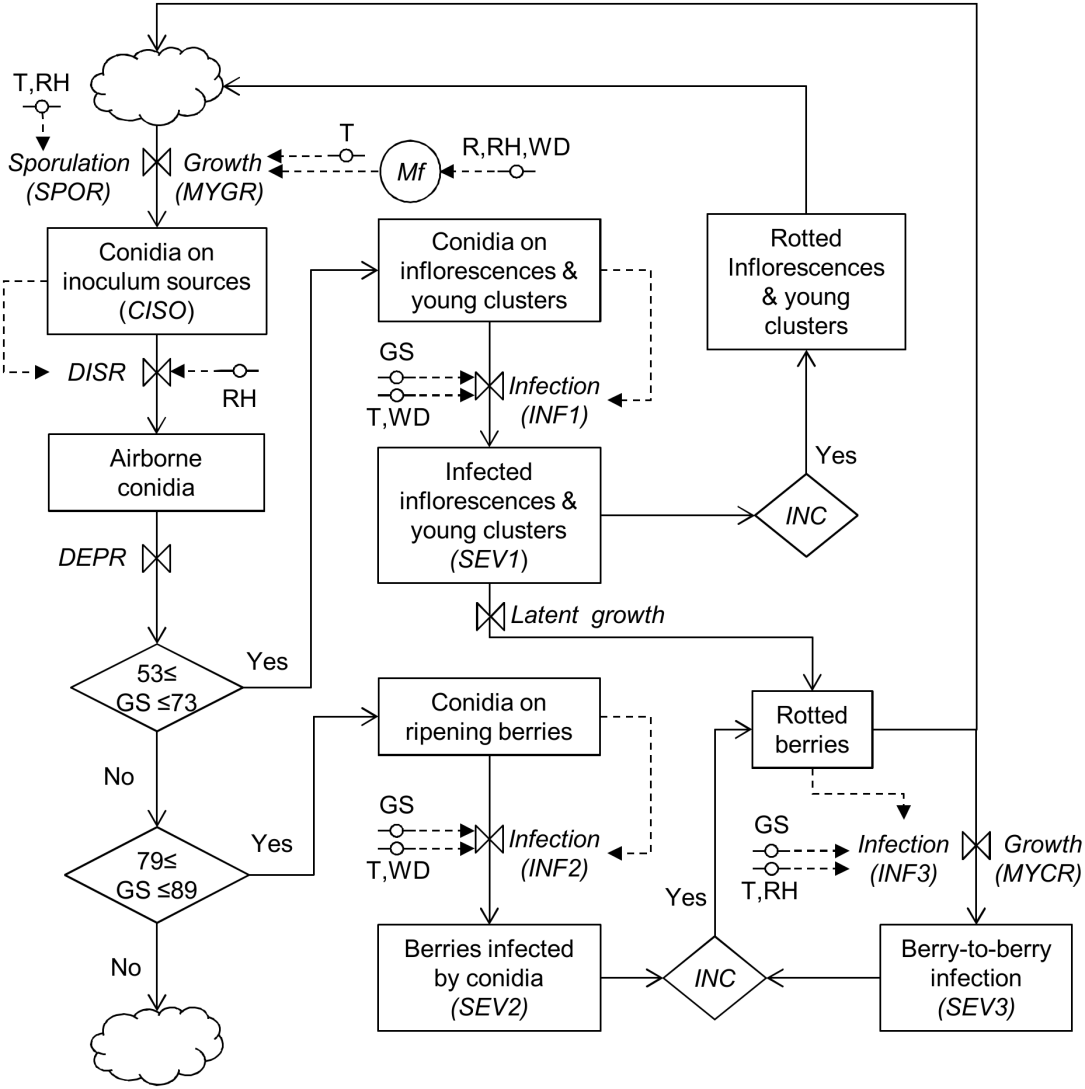
Relational diagram of the model simulating the life cycle of *Botrytis cinerea*. Legend: boxes are state variables; line arrows show fluxes and direction of changes; valves define rates regulating these fluxes; diamonds show switches (i.e., conditions that open or close a flux); circles crossed by a line show parameters and external variables; dotted arrows show fluxes and direction of information from external variables to rates; circles are intermediate variables; clouds indicate outgoing variables. See Table 1 for acronym explanations.

The model was elaborated according to the principles of “systems analysis” [69]. The *B*. *cinerea* life cycle was divided into different state variables, and changes from one state to the following one were determined by rate variables depending on environmental conditions and host growth stages, as shown in the relational diagram of the model (Fig 1 and Table 1). The model calculations begin when grape inflorescences are clearly visible and ends when berries are ripe for harvesting, with a time step of 1 day.

**Table 1:** List of variables, rates, and parameters used in the model describing the life cycle of *Botrytis cinerea* on grapevines.

Acronym	Description	Unit
*CISO*	Relative abundance of conidia on inoculum sources	Number (0 to 1)
*MYGR*	Mycelium growth rate	Number (0 to 1)
*SPOR*	Sporulation growth rate	Number (0 to 1)
*DISR*	Dispersion rate	Number (set at 1)
*DEPR*	Deposition rate	Number (set at 1)
*INC*	Incubation period	-^a^
*INF1*	Infection rate on inflorescences and young clusters	Number (0 to 1)
*SEV1*	Accumulated value of relative infection severity of the inflorescences and young clusters	Number
*INF2*	Rate for conidial infection on ripening berries	Number (0 to 1)
*SEV2*	Accumulated value of relative infection severity for conidial infection on ripening berries	Number
*INF3*	Rate for berry-to-berry infection on ripening berries	Number (0 to 1)
*SEV3*	Accumulated value of relative infection severity for berry-to-berry infection on ripening berries	Number
T	Air temperature	°C
RH	Relative humidity	%
R	Rainfall	mm
WD	Wetness duration	h
GS	Growth stage of the host based on the scale of Lorenz et al. [31]	Number (0–99)
Mf	Factor accounting for moisture	Number (0 to 24)

^a^ Incubation period was not considered in this model.

### Model description

The model considers that multiple inoculum sources are present in the vineyard all season. These inoculum sources include dead, infested plant material; affected inflorescences and young berries; infested floral debris; and affected ripening berries (Fig 1). The relative abundance of conidia on these sources (*CISO*) on any day *i* of the grape-growing season depends on the rate at which the mycelium grows and saprophytically colonises the source tissue (*MYGR*) and on the rate of spore production (*SPOR*), as follows:

$$CISO_{i}=\frac{\sum_{i-6}^{i}MYGR_{n}\times SPOR_{n}}{7}$$ (1)

with

$$MYGR_{n}={\left(3.78\times Teq_{n}^{0.9}\times (1-Teq_{n})\right)}^{0.475}\times Mf_{n}$$ (2)

$$SPOR_{n}={\left(3.7\times Teq_{n}^{0.9}\times (1-Teq_{n})\right)}^{10.49}\times \left(-3.595+0.097\times RH_{n}-0.0005\times RH_{n}^{2}\right)$$ (3)

In these equations, *Teq* = temperature equivalent in the form *Teq* = (*T—Tmin*) / (*Tmax—Tmin*); with *T* = daily average temperature (°C); *Tmin* = minimum temperature for mycelial growth or sporulation (0°C); *Tmax* = maximum temperature for mycelial growth (40°C) or sporulation (35°C); and *Mf* = factor accounting for the effect of moisture of the medium (e.g., bunch trash) on mycelial growth. With respect to *Mf*, *B*. *cinerea* mycelium grows quickly when the water activity of the medium is a_w_≥0.95 (unpublished data), which corresponds to residue moisture greater than 60 to 80% depending on temperature and sorption or desorption conditions [70]. We therefore assumed that residues contain sufficient humidity for mycelial growth in the hours of the day when rain, R≥0.2 mm or wetness duration, WD≥30 min or RH (daily average relative humidity)≥90% (i.e., moist hours); therefore, *Mf* = number of moist hours/24.

Eqs (2) and (3) were derived from Ciliberti et al. [14] and Ciliberti et al. [30], respectively. These equations were averaged over a 7-day period because researchers previously reported that this is the duration for sporulation of a single lesion [30,71].

The model assumes that, on any day, there are favourable conditions for conidia to disperse and settle on host plant surfaces, so that *DISRi* = 1 and *DEPRi* = 1 (Fig 1). This assumption is consistent with our knowledge about *B*. *cinerea* epidemiology, as described in the section “Life cycle of *Botrytis cinerea*”.

The model considers two main infection periods (or infection windows) corresponding to two grape-growing periods (Fig 1). The first infection window occurs between “inflorescences clearly visible” (stage 53 of the scale of Lorenz et al. [31] and “berries groat-sized, bunches begin to hang” (stage 73); in this period the model calculates infection by conidia on inflorescences and young clusters (pathway I and II). The second infection window occurs between “majority of berries touching” (stage 79) and “berries are ripe for harvest” (stage 89); in this period, the model calculates infection on ripening clusters by conidia (pathway Va) and berry-to-berry infection by aerial mycelium (pathway Vb). Compared to the relational diagram of Fig 1, the model described in this paper does not consider latent infections and incubation periods (*latent growth* and *INC* in Fig 1) because there is insufficient quantitative information about the effect of weather and host conditions on latency and incubation.

In the first infection window (stages 53 to 73), the model calculates an infection rate on inflorescences and young clusters (*INF*1) as:

$$INF1_{i}=\frac{{\left(3.56\times Teq_{i}^{0.99}\times (1-Teq_{i})\right)}^{0.71}}{\left(1+e^{\left(1.85-0.19\times WD_{i}\right)}\right)}\times SUS1$$ (4)

where *Teq* = temperature equivalent as described for eq (2), with *Tmin* = 0°C and *Tmax* = 35°C;

*WD* = wetness duration (in hours); and

$$SUS1_{i}=-379.09\times {\left(\frac{GS_{i}}{100}\right)}^{3}+671.25\times {\left(\frac{GS_{i}}{100}\right)}^{2}-390.33\times \left(\frac{GS_{i}}{100}\right)+75.209$$ (5)

In the latter equation, *SUS1* = relative susceptibility of the inflorescences and young clusters, and *GS* = growth stage of the plant based on the stages of the scale of Lorenz et al. [31].

Eq (4) was developed by Ciliberti et al. [14]. Eq (5) was developed by using data from Ciliberti et al. [14] as described in S1 Appendix.

Relative infection severity in the first infection window is then calculated as:

$$RIS1_{i}=CISO_{i}\;\times \;INF1_{i}$$ (6)

In the second infection window (stages 79 to 89), the model calculates two infection rates on ripening berries: one for conidial infection (*INF*2) and another for berry-to-berry infection (*INF*3) (Fig 1).

Infection rate for conidial infection is calculated as follows:

$$INF2_{i}={\left(6.416\times Teq_{i}^{1.292}\times \left(1-Teq_{i}\right)\right)}^{0.469}\times e^{-2.3\times e^{\left(-0.048\times WD\right)}}\times SUS2_{i}$$ (7)

where *Teq* = temperature equivalent as described for eq (2) with *Tmin* = 0°C and *Tmax* = 35°C, and

$$SUS2_{i}=5\times 10^{-17}\times e^{0.4219\times GS_{i}}$$ (8)

In the latter equation, *SUS2* = relative susceptibility of the ripening berries to *B*. *cinerea* conidia infection, and GS = growth stage of the plant based on the scale of Lorenz et al. [31].

Eq (7) was developed by Ciliberti et al. [17], and eq (8) was developed based on data from Deytieux-belleau et al. [18] as explained in S1 Appendix.

Relative infection severity for conidial infection is then calculated as follows:

$$RIS2_{i}=CISO_{i}\times INF2_{i}$$ (9)

Infection rate for berry-to-berry infection during the second infection window is calculated as follows:

$$INF3_{i}=\frac{{\left(7.75\times Teq_{i}^{2.14}\times (1-Teq_{i})\right)}^{0.469}}{1+e^{\left(35.36-40.26\times \frac{RH_{i}}{100}\right)}}\times SUS3_{i}$$ (10)

where *Teq* = temperature equivalent as described in eq (2) with *Tmin* = 0, and *Tmax* = 30°C, and

$$SUS3_{i}=0.0546\times GS_{i}-3.87\;\left(\text{when }SUS3_{i}>1,\text{ then }SUS3_{i}=1\right)$$ (11)

In the latter equation, *SUS3* = relative susceptibility of the ripening berries to *B*. *cinerea* mycelium infection, and GS = growth stage of the plant based on the scale of Lorenz et al. [31].

Eq (10) was developed by Ciliberti et al. [17], and eq (11) was developed based on one experiment from Ciliberti et al. [17] as described in S1 Appendix.

Relative infection severity for berry-to-berry infection is then calculated as follows:

$$RIS3_{i}=INF3_{i}\times MYGR_{i}$$ (12)

Daily values of relative infection severity (i.e., *RIS1*, *RIS2*, and *RIS3*) are finally accumulated over the time of the infection window that they refer to. These accumulated values produce new variables (named *SEV1*, *SEV2*, and *SEV3*, respectively), which provide a picture of the total risk of infection.

**Fig 2:**
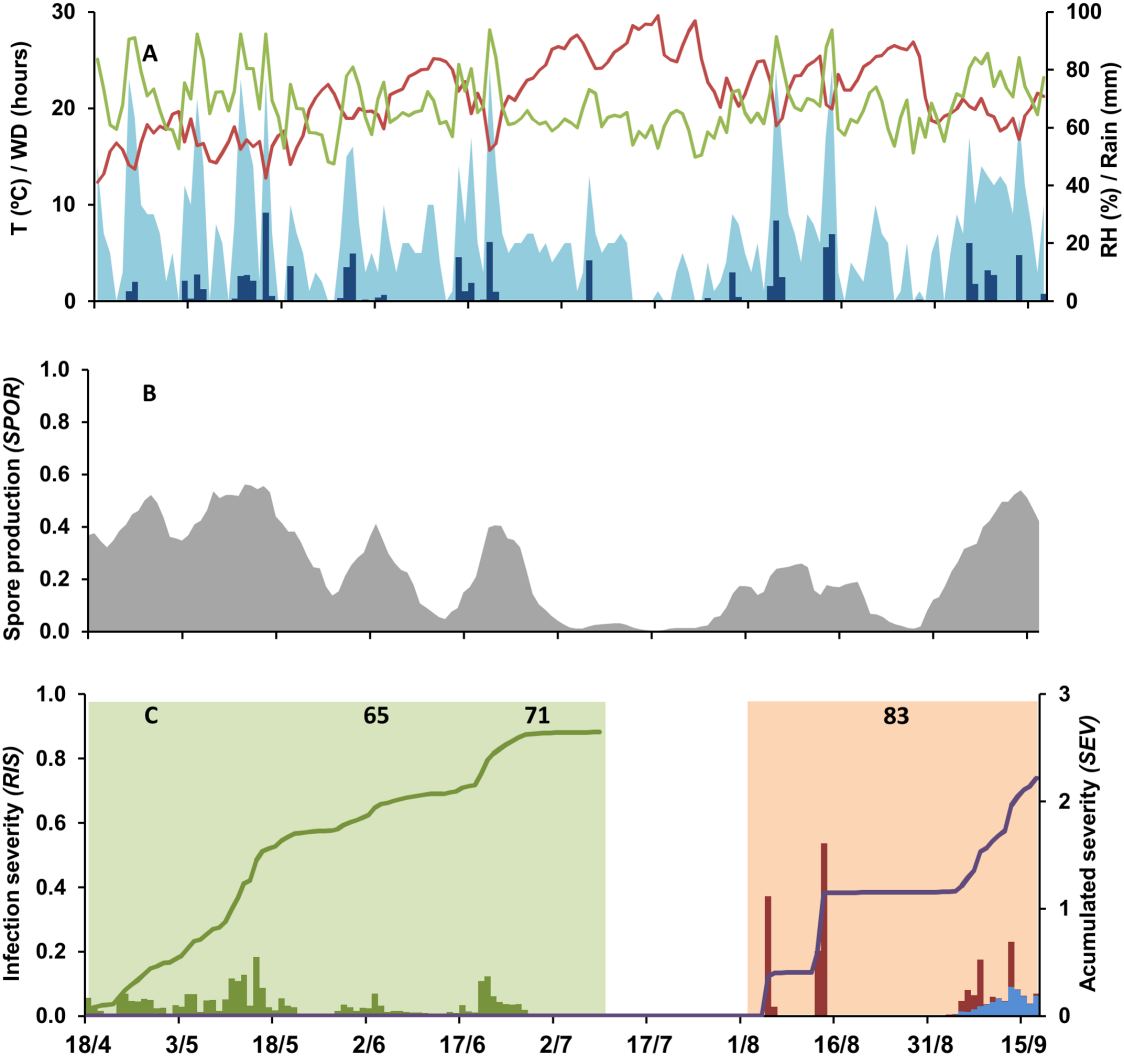
Weather data and model output at Conselice in 2010 (CO-10). A: daily data of temperature (red line, T in °C), relative humidity (green line, RH in %), rain (blue bars, in mm), and wetness duration (light blue area, WD in hours); B: predicted rate of spore production (*SPOR*) by *Botrytis cinerea*; C: predicted relative infection severity (*RIS*) on inflorescences and young clusters by conidia (*RIS1*; green bars) during the first infection period (green area), and on ripening berries by conidia (*RIS2*; blue bars) and mycelium (berry-to-berry infection) (*RIS3*; red bars) during the second infection period (orange area). Lines indicate the accumulated values of *RIS1* (*SEV1*; green line) and of *RIS2+RIS3* (*SEV2+SEV3*; purple line). Numbers in C indicate vine growth stages of full flowering (stage 65 of the scale of Lorenz et al. [31]), fruit set (stage 71), and berries developing (stage 83). The first infection window extends from inflorescences clearly visible” (stage 53) to “berries groat-sized, bunches begin to hang” (stage 73). The second infection window extends from “majority of berries touching” (stage 79) to “berries are ripe for harvest” (stage 89).

**Fig 3:**
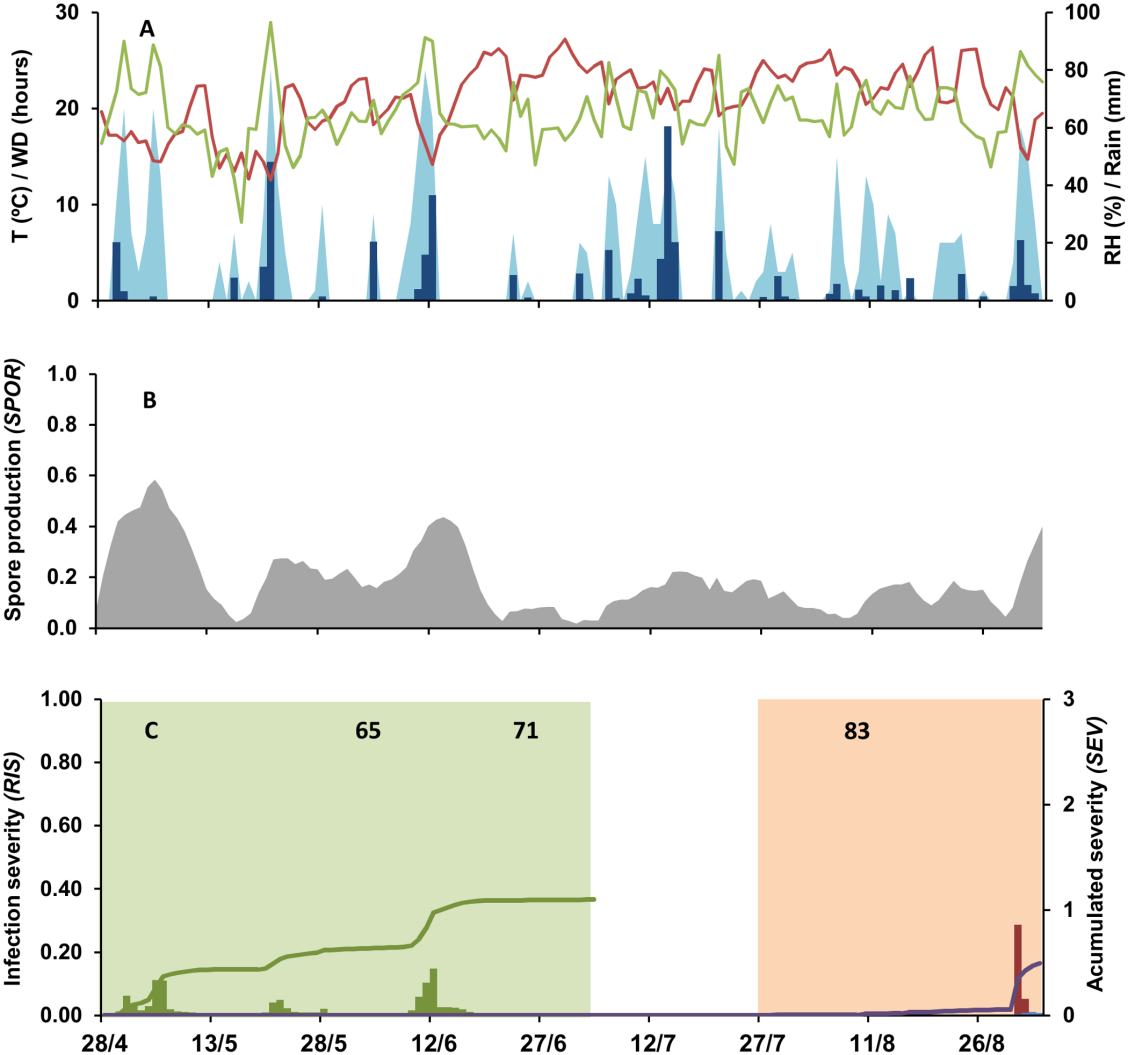
Weather data and model output at S. Michelle in 2012 (SM-12). A: daily data of temperature (red line, T in °C), relative humidity (green line, RH in %), rain (blue bars, in mm), and wetness duration (light blue area, WD in hours); B: predicted rate of spore production (*SPOR*) by *Botrytis cinerea*; C: predicted relative infection severity (*RIS*) on inflorescences and young clusters by conidia (*RIS1*; green bars) during the first infection period (green area), and on ripening berries by conidia (*RIS2*; blue bars) and mycelium (berry-to-berry infection) (*RIS3*; red bars) during the second infection period (orange area). Lines indicate the accumulated value of *RIS1* (*SEV1*; green line) and of *RIS2+RIS3* (*SEV2+SEV3*; purple line). Numbers in C indicate vine growth stages of full flowering (stage 65 of the scale of Lorenz et al. [31]), fruit set (stage 71), and berries developing (stage 83). The first infection window extends from “inflorescences clearly visible” (stage 53) to “berries groat-sized, bunches begin to hang” (stage 73). The second infection window extends from “majority of berries touching” (stage 79) to “berries are ripe for harvest” (stage 89).

Two examples of model output are shown in Figs 2 and 3 (epidemics at CO-09 and SM-12 in Table 2, respectively) as follows: weather data are shown in Figs 2A and 3A, sporulation on inoculum sources in Figs 2B and 3B, and the daily and accumulated values of relative infection severity in Figs 2C and 3C. The considered period (i.e., between grape growth stages 53 and 89) was 153 days long at CO-10 and 129 days long at SM-12. At both locations, > 350 mm of rain fell in this period. This rain was more regularly distributed at CO-10 than at SM-12, and the number of hours with wetness was higher at CO-10 than at SM-12 (1060 vs. 530 h, respectively), especially during the first infection window (619 h for CO-10 and 254 h for SM-12) (Figs 2A and 3A). As a consequence, sporulation was abundant and regular at CO-10 during the first infection window, and a constant increase of *RIS1* was observed, with an accumulated value *SEV1* = 2.64 at the end of this period (Fig 2B and 2C). In the same period at SM-12, the model predicted lower levels of sporulation and lower values of *RIS1*, with only two peaks at the end of April and the beginning of June, resulting in an accumulated value *SEV1* = 1.10 at the end of the first infection period (Fig 3B and 3C). During the second infection period, the wetness duration decreased in both vineyards, and lower values of sporulation were predicted (Figs 2 and 3). At CO-10, high values of *RIS2* and *RIS3* were predicted only in early August and in the last 2 weeks before harvest. At SM-12, the model predicted favourable conditions for *B*. *cinerea* 1 week before harvest. Consequently, the values of *SEV2*+*SEV3* were higher for CO-10 (2.61) than for SM-12 (0.5).

**Table 2:** Summary characteristics (location, year, cultivar, and training system) of the vineyards used for validation of the *Botrytis cinerea* model. Also indicated are the observed incidence and severity of Botrytis bunch at maturity and the classification of the epidemics.

Code of epidemics	Vineyard locality (country)^a^	Year	Cultivar	Training system	Observed incidence (%)^b^	Observed severity (%)^c^	Observed epidemic group^d^
BAG-09	Bagnacavallo (IT)	2009	Trebbiano Romagnolo	Casarsa	64.5	3.3	Intermediate
BAG-11	Bagnacavallo (IT)	2011	Trebbiano Romagnolo	Casarsa	35.0	4.6	Intermediate
BAG-12	Bagnacavallo (IT)	2012	Trebbiano Romagnolo	Casarsa	78.0	3.3	Severe
CA-13	Camerlona (IT)	2013	Trebbiano Romagnolo	Casarsa	68.5	6.8	Intermediate
CO-09	Conselice (IT)	2009	Trebbiano Romagnolo	Guyot	85.5	24.9	Severe
CO-10	Conselice (IT)	2010	Trebbiano Romagnolo	Guyot	81.8	15.9	Severe
CO-11	Conselice (IT)	2011	Trebbiano Romagnolo	Guyot	54.5	5.8	Intermediate
CO-12	Conselice (IT)	2012	Trebbiano Romagnolo	Guyot	94.5	48.0	Severe
CO-13	Conselice (IT)	2013	Trebbiano Romagnolo	Guyot	80.8	15.1	Severe
COT-13	Barbiano di Cotignola (IT)	2013	Trebbiano Romagnolo	Casarsa	65.0	9.7	Intermediate
RAV-12	Ravenna (IT)	2012	Trebbiano Romagnolo	Casarsa	78.0	15.0	Severe
RUS-10	Godo di russi (IT)	2010	Trebbiano Romagnolo	Casarsa	56.7	5.5	Intermediate
MT-13	Montepaldi (IT)	2013	Trebbiano Toscno	S. Cordon	60.0	13.0	Intermediate
SJ-14	Saint Julien (FR)	2014	Merlot	Guyot	27.4	1.6	Intermediate
SM-12	S. Michelle (IT)	2012	Pinot Gris	Guyot	2.2	0.2	Mild
SM-13	S. Michelle (IT)	2013	Pinot Gris	Guyot	0.0	0.0	Mild
V-14	Villanave d’Ordon (FR)	2014	Merlot	Guyot	36.7	1.6	Intermediate
VI-14	Villanova di Ravenna (IT)	2014	Trebbiano Romagnolo	Casarsa	79.5	12.6	Severe
Z-11	Ziano (IT)	2011	Barbera	Guyot	37.5	13.5	Intermediate
Z-12	Ziano (IT)	2012	Barbera	Guyot	1.3	4.4	Mild
Z-13	Ziano (IT)	2013	Barbera	Guyot	0.0	0.0	Mild

^a^ Country code: IT = Italy; FR = France.

^b^ Disease incidence assessed as the percentage of bunches with Botrytis rot at maturity.

^c^ Disease severity assessed as the percentage of the bunch surface affected by Botrytis rot at maturity.

^d^ Epidemic group: severe (incidence ≥ 75%; severity ≥ 15%); intermediate (74 ≥ incidence ≥ 25%; severity < 15%); mild (incidence < 24%; severity < 5%).

## Model validation

### Field assays

The model output was validated against real data obtained from 2009 to 2014 from 21 Botrytis bunch rot epidemics in untreated plots in 12 experimental vineyards. These data have not been previously used for model development. Ten of these vineyards were in Italy, and two were in France (Table 2), under Cfb and Cfc climate types, respectively (C = warm temperate; f = fully humid precipitation; b = warm summer; c = cold summer) [72]. The vineyards were cropped with grape varieties highly susceptible to *B*. *cinerea* [48,57,73] and were managed as usual for the viticultural area, with the exception that no fungicides were used to control *B*. *cinerea*. Standard, electronic weather stations were installed in the vineyard borders (with sensors at 1.5 m above the ground) to measure temperature, relative humidity, wetness duration, and rainfall on an hourly basis. Growth stages of vines were periodically assessed in the vineyards according of the scale of Lorenz et al. [31]. Weather and vine growth stages were used as model inputs to calculate model output (i.e., *SEV1*, *SEV2*, and *SEV3*).

At full ripening (growth stage 89), disease was assessed on a minimum of 100 random bunches per plot (on at least 20 vines per plot) and in at least four replicate plots per vineyard. Disease incidence (DI) and severity (DS) were visually assessed as the percentage of bunches with Botrytis rot and as the percentage of the surface of the bunch affected by rot and showing typical *B*. *cinerea* sporulation, respectively. Based on disease assessment, the 21 epidemics were placed into three groups based on the following classification rules: severe, DI ≥ 75% (and DS ≥ 15%); intermediate, 74 ≥ DI ≥ 25% (and DS < 15%); and mild, DI < 24% (and DS < 5%). Threshold values for these groups were selected based on the literature and personal communications from grape growers, advisors, and wineries. Epidemic classified as mild (DS<5%) result in yield losses the growers are willing to accept; disease severities of 3% [74], 5% [75] or 6% [76] were considered thresholds for fungicide application. The need of applying fungicides with intermediate epidemics (DS<15%) depends on the risk perception by the grower [77]; risky growers prefer to avoid treatments as they consider the risk of yield losses as acceptable. Finally, no growers renounce to protect the crop when the expected DS is ≥ 15%.

### Ethics statements

The owners of the vineyards used for model validation gave permission to conduct the study on these sites.

### Data analysis

A discriminant function analysis (DFA) was performed to determine whether *SEV1*, *SEV2*, and *SEV3* (discriminating variables) were effective in predicting whether epidemics fell into one of three groups: severe, intermediate, or mild [78–81]. The analysis created two linear combinations of discriminant functions in the form: *F*_*n*_ = *a*_*n*_ + *b*_*n*,*1*_ *·ln*(*SEV1*+1) + *b*_*n*,*2*_ *·ln*(*SEV2*+*SEV3*+1), where n = 1 or 2 for the two discriminant functions, respectively. Briefly, the procedure automatically selected a first function (*F*_*1*_) that was able to separate the groups as much as possible; a second function (*F*_*2*_) was then selected that was both uncorrelated with the first function and that provided as much further separation as possible. For each epidemic group, the centroid (i.e., the mean value of each group) was calculated.

The two functions were used to: (i) locate the cases (epidemics) into the *F*_*1*_ –*F*_*2*_ space based on the coordinates obtained by solving the two discriminant functions; (ii) define the case distance from the centroid of each group (as the Mahalanobis distance; [81]); (iii) determine the probability that each case belonged to each of the three groups and determine group membership based on these probabilities; and (iv) obtain information on the effect of the discriminant variables on group membership. For (iii), prior probability of membership was set proportional to the number of epidemics in each group (i.e., 0.19 for mild, 0.48 for intermediate, and 0.33 for severe epidemics). For (iv), the canonical coefficients (CCs), standardized canonical coefficients (SCCs), and the correlation coefficients (COCs) of the two functions were calculated. The magnitude of CCs and SCCs is an indicator of the weight of each variable in each of the discriminant functions; COCs indicate the discriminant power of each variable in these functions.

The Wilks' lambda was calculated as a measure of how well each function separated cases into the three groups; it is equal to the proportion of the total variance in the discriminant scores not explained by differences among the groups. Lambda ranges from 0 to 1, with 0 indicating that group means differ (i.e., that the function strongly differentiates groups), and 1 indicating that all group means are the same. A chi-square statistic was used to test the hypothesis that the means of the functions listed were equal across groups (a small significance value for this test indicates that the discriminant function does better than chance at separating the groups) [80].

DFA was performed using the discriminant procedure of the statistical software SPSS (ver. 21.0, IBM SPSS Statistics, IBM Corp., New York, USA).

### Evaluation of model predictions during the season

DFA enables prediction of new cases based on the previously established structure [82]. In this section, DFA was evaluated to anticipate the prediction of the final severity of the *B*. *cinerea* epidemic (i.e., mild, intermediate, or severe) by using the daily model output, i.e., the daily values of *SEV1*, *SEV2*, and *SEV3*.

The daily model outputs (the natural logarithms of daily values of *SEV1* and *SEV2*+*SEV3*) were used to solve the two canonical discriminant functions (*F*_*1*_ and *F*_*2*_) by using the non-standardized canonical coefficients calculated in the previous section. This allowed us to locate the cases in the *F*_*1*_ –*F*_*2*_ space and to calculate the standardized distance (*D*_*uj*_) of each case (*u*) to the centroid of each group (*j*) (i.e., mild, intermediate, or severe) by using the Mahalanobis distance [81]. Because the DFA considered three groups, a multivariate approach was used: the different cases and centroids were represented in the *F*_*1*_ –*F*_*2*_ space as vectors, and matrix operations were used to resolve the equations [81]. The Mahalanobis distance was calculated in the form:

$$D_{uj}={\left[{\left(x_{u}-\sigma_{j}\right)}^{\prime }\sum_{j}^{-1}\left(x_{u}-\sigma_{j}\right)\right]}^{1/2}$$

where *x*_*u*_ is the vector of case *u*, *σ*_*j*_ is the vector of the centroid of population *j*, and $\sum_{j}^{-1}$ is the inverse of the covariance matrix of population *j*.

To assign each case *u* to one of the *j* groups, the posterior probability of membership *P*(*j*|*x*_*u*_) was calculated as follows [81]:

$$P\left(j|x_{u}\right)=\frac{\alpha_{j}\times f\left(x_{u}|j\right)}{\sum_{j}\alpha_{j}\times f\left(x_{u}|j\right)}$$

Where *f*(*x*_*u*_|*j*) is the likelihood of *x*_*u*_ = *j*, and *α*_*j*_ is the prior probability of group membership (i.e., the proportion of the 21 vineyards used for model validation belonging to each epidemic group as described in the previous section). Assuming a normal distribution for the population of each *j* group, we defined the density value *f*(*x*_*u*_|*j*) with a multivariate approach as described by Huberty and Olejnik [81]:

$$f\left(x_{u}|j\right)=\frac{1}{\sqrt{2\pi }\sqrt{\left|\sum_{j}\right|}}e^{\left(-\frac{1}{2}D_{uj}^{2}\right)}$$

Where |∑_*j*_| is the determinant of the covariance matrix of population, and *D*_*uj*_ is the Mahalanobis distance of each case *u* to the centroid of each group *j*.

With this procedure, the probability that the *B*. *cinerea* epidemic would be mild (*P*_*mild*_), intermediate (*P*_*int*_), or severe (*P*_*sev*_) was calculated for each day and for each of the 21 epidemics described in the “field assays” section. For each day between growth stages 53 and 89, each epidemic was assigned to one of the three severity groups by using the maximum-likelihood principle, i.e., by selecting the group with the highest probability of occurrence [81].

### Results of Model Validation

Of the 21 *B*. *cinerea* epidemics, four were mild (with average disease incidence = 0.9±0.54, and average severity = 1.2 ± 1.08), 10 were intermediate (with disease incidence and severity equal to 50.6 ± 4.7 and 6.6 ± 1.4, respectively), and seven were severe (with disease incidence and severity of 82.6 ± 2.2 and 19.3 ± 5.3, respectively) (Table 2).

**Fig 4:**
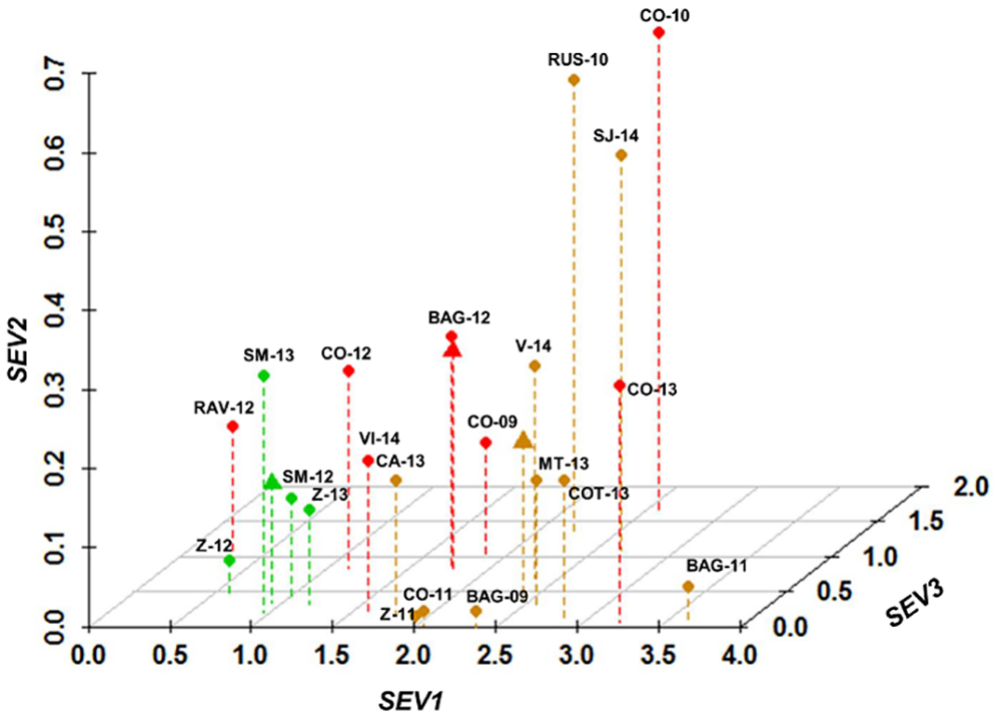
Model output (i.e., *SEV1*, *SEV2*, and *SEV3*) for 21 *Botrytis cinerea* epidemics. The 21 epidemics are coded as in Table 2; their symbols (dots) are green, orange or red based on Botrytis bunch rot incidence and severity as indicated in Table 2 (green, orange, and red indicate mild, intermediate, and severe epidemics, respectively). Triangles represent the average of each group. *SEV1* = relative severity of the infections caused by conidia and accumulated over the first infection period (“inflorescences clearly visible” to “berries groat-sized, bunches begin to hang”); *SEV2* = relative severity of the infections caused by conidia and accumulated over the second infection period (“majority of berries touching” to “berries are ripe for harvest”); and *SEV3* = relative severity of the infections caused by mycelium (berry-to-berry infection) and accumulated over the second infection period.

Model predictions for these epidemics are shown in Fig 4. For mild epidemics, the average values of *SEV1*, *SEV2*, and *SEV3* were 0.94 ± 0.13, 0.15 ± 0.06, and 0.34 ± 0.06, respectively. The lowest values were calculated at Z-12 (Fig 4), where the Botrytis bunch rot incidence at harvest was 1.3% (Table 2). Average values of *SEV1* were higher for intermediate (2.46 ± 0.17) and severe epidemics (1.81 ± 0.35) than for mild epidemics (0.94 ± 0.13). Values of *SEV2* and *SEV3* were higher for severe epidemics (0.28 ± 0.06 and 0.76 ± 0.19, respectively) than for intermediate epidemics (0.20 ± 0.06 and 0.37 ± 0.16, respectively). The highest values of *SEV2* and *SEV3* were calculated at C0-10, where Botrytis bunch rot incidence was 81.8%, and severity was 15.9% at harvest (Table 2).

**Fig 5:**
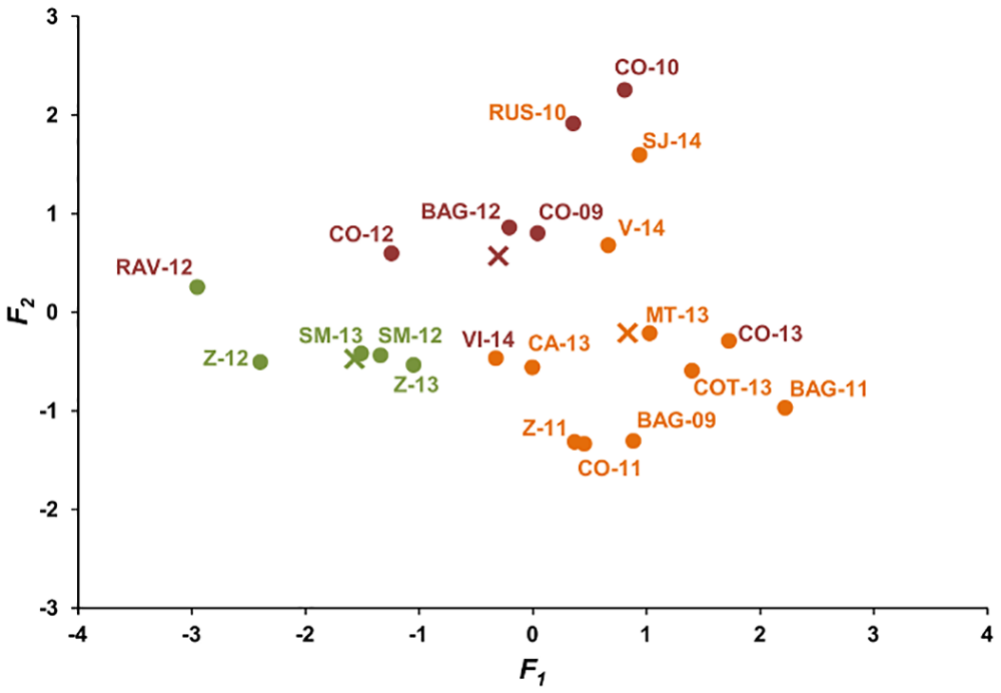
Assignment of 21 *Botrytis cinerea* epidemics to three severity groups based on the discriminant function analysis. The 21 epidemics are coded as in Table 2; the color of the code indicates the severity of Botrytis bunch rot as assessed in the vineyard at harvest (see Table 2). The color of the dot indicates group assignment based on DFA. For both codes and dots, green, orange, and red indicate mild, intermediate, and severe epidemics, respectively. Crosses represent the centroids of each group based on DFA. Coefficients of the canonical discriminant functions are shown in Table 6.

DFA correctly classified 17 of the 21 epidemics (i.e., 81% of correct classification) (Table 3). In three of the four misclassifications, the real epidemic was more severe than the predicted one: epidemics at RAV-12, VI-14, and CO-13 were all severe based on disease assessment but were classified as mild or intermediate (Fig 5). In contrast, the epidemic at RUS-10 was intermediate but was classified as severe by the DFA (Fig 5). Using cross-validation, 15 of the 21 epidemics (71.4%) were correctly classified (Table 3).

**Table 3:** Grouping of 21 *Botrytis cinerea* epidemics based on disease assessment in vineyards and as predicted based on the discriminant function analysis (DFA) using model output as discriminant variables.

		Predicted group membership^c^		
		Mild	Intermediate	Severe
**Real group membership** ^**a**^	**Mild**	4	0	0
	**Intermediate**	0	9	1
	**Severe**	1	2	4
**Cross-validated** ^**b**^	**Mild**	4	0	0
	**Intermediate**	0	8	2
	**Severe**	1	3	3

^a^ Real group membership is based on disease incidence (DI) and severity (DS) of Botrytis rot assessed in the vineyards at maturity. Mild, DI < 24% and DS < 5%; intermediate 74 ≥ DI ≥ 25% and DS < 15%; severe, DI ≥ 75% and DS ≥ 15%.

^b^ In cross validation, each case is classified by the functions derived from all cases other than that case.

^c^ Group membership predicted by the DFA.

In this separation of epidemics into three groups, *F*_*1*_ accounted for 82.2% of the variation and *F*_*2*_ accounted for the remaining 17.2% (Table 4); canonical correlation coefficients (Table 4) and the significance of Wilks’ lambda tests (Table 5) all indicated that *F*_*1*_ had greater effect on group separation than *F2*. Canonical coefficients (CC), standardized canonical coefficients (SCC), and correlation coefficients (COC) collectively indicated that *SEV1* had a major effect in distinguishing the groups in *F*_*1*_ and that *SEV2+SEV3* had a major effect in distinguishing the groups in *F*_*2*_ (Table 6).

**Table 4:** Statistics of the discriminant function analysis used to classify 21 *Botrytis cinerea* epidemics based on model output.

Function	Eigenvalue	% of Variance^a^	Canonical correlation^b^
***F***_***1***_	0.971	82.2	0.702
***F***_***2***_	0.202	17.2	0.410

^a^ Proportion of total variance explained by each discriminant function.

^b^ Multiple correlation between the discriminant variables and each discriminant function.

**Table 5:** Significance of the discriminant function analysis used to classify 21 *Botrytis cinerea* epidemics based on model output.

Test of functions	Wilks’ Lambda^a^	Chi-square^b^	df	Significance^c^
***F***_***1***_ **through *F***_***2***_	0.422	15.087	4	0.005
***F***_***2***_	0.832	3.216	1	0.073

^a^ Proportion of the total variance in the discriminant scores not explained by differences among the groups: 0 indicates that group means differ, and 1 indicates that all group means are the same.

^b^ Chi-square statistic used to test the hypothesis that the means of the functions listed are equal across groups.

^c^ A small significance value indicates that the discriminant function does better than chance at separating the groups.

**Table 6:** Coefficients for each discriminant variable in each canonical function (*F*_*1*_ or *F*_*2*_) obtained in the discriminant analysis used to classify 21 *Botrytis cinerea* epidemics based on model output.

	Canonical coefficient^b^		Standardized canonical coefficient^c^		Correlation coefficient^d^	
Discriminant variables^a^	*F*_*1*_	*F*_*2*_	*F*_*1*_	*F*_*2*_	*F*_*1*_	*F*_*2*_
***SEV1***	4.180	0.595	0.991	0.141	0.995*	0.101
***SEV2* + *SEV3***	-0.310	3.067	-0.101	0.996	-0.141	0.990*
**Constant**	-4.191	-2.090				

^a^ *SEV1* = accumulated value of relative infection severity of the inflorescences and young clusters; *SEV2* = accumulated value of relative infection severity for conidial infection on ripening berries; *SEV3* = accumulated value of relative infection severity for berry-to-berry infection on ripening berries.

^b^ Coefficients of the discriminant functions. The discriminant function take the form: *F* = *a* + *b*_*1*_ ln(*SEV1*+1) + *b*_*2*_ ln(*SEV2*+*SEV3*+1), where *a* is a constant and *b*_*n*_ are the canonical coefficients.

^c^ The standardized canonical coefficient is an indicator of the weight of each variable in each function.

^d^ The correlation coefficient indicates the discriminant power of each variable in each function.

* indicate largest absolute correlation between each variable and any discriminant function. Variables with correlation coefficient ≥ 0.3 are interpreted as important [79].

**Fig 6:**
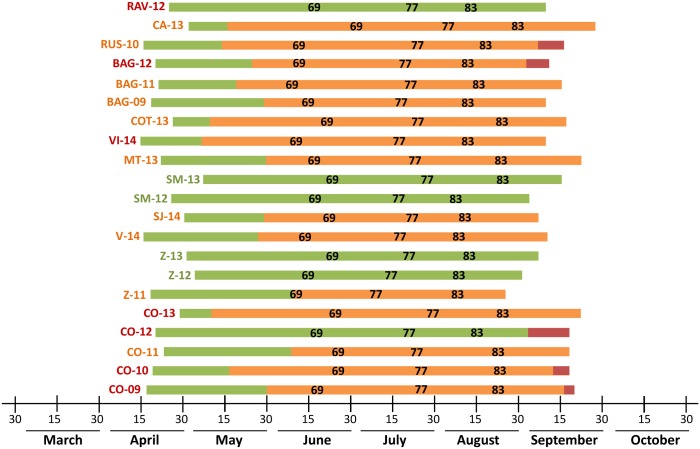
Assignment of 21 *Botrytis cinerea* epidemics to mild, intermediate, and severe groups based on model output. Discriminant function analysis (DFA) was calculated daily between “inflorescences clearly visible” (growth stage 53 of Lorenz et al. [31]) and “berries ripe for harvest” (stage 89). The colors of the 21 horizontal bars indicate the daily assignment to epidemic groups based on DFA. The 21 epidemics are also coded on the left of each horizontal bar based on the severity of Botrytis bunch rot as observed in the vineyard at harvest and as indicated in Table 2. For both codes and bars, green, orange, and red indicate mild, intermediate, and severe epidemics, respectively. For example, the epidemic at BAG-12 was severe at harvest based on observation and progressed from mild to intermediate to severe based on DFA. Numbers in bars indicate the critical growth stages for fungicide applications; end of flowering (stage 69); pre-bunch closure (stage 77); veraison (stage 83).

Fig 6 shows the daily changes in the assignment of the 21 *B*. *cinerea* epidemics to one of three categories (mild, intermediate, or severe) based on the DFA of the daily model outputs (i.e., daily values of *SEV1*, *SEV2*, and *SEV3*). Five of these epidemics (at Z-12, Z-13, SM-12, SM-13, and RAV-12) remaining mild all season. The other epidemics were assigned as intermediate starting from the first week of May (at CO-13, VI-14, and COT-13) to the first of June (CO-11 and Z-11), which was between 47 and 13 days before vine growth stage 69 (i.e., end of flowering) (Fig 6). For the epidemic at Z-11, the assignment of intermediate severity occurred at stage 69 (Fig 6). The change from mild to intermediate epidemics always occurred during the first infection period of the model (i.e., growth stages 53 to 73) (Fig 6), when the model calculates *SEV1*. In the five cases in which the epidemic was assigned to the group of severe epidemics (C-09, C-10, C-12, BAG-12, and RUS-10), the change from intermediate to severe occurred after growth stage 83 (veraison), between 3 days (CO-09) and 8 days (CO-12) before growth stage 89 (berries ripe for harvest) (Fig 6). At CO-12, the classification jumped from mild to severe.

## Discussion

In this work, a new, mechanistic model for *B*. *cinerea* infections was elaborated [83]. In creating this model, we divided the *B*. *cinerea* life cycle into different state variables, and changes from one state to the following state were determined by rate variables that depended on environmental conditions and that were represented in mathematical equations. Equations were previously published on the effects of environmental variables and grape phenology on *B*. *cinerea* sporulation and on *B*. *cinerea* infection of inflorescences, young berries clusters, and mature berries [14,17,30]. The ability of these equations to correctly reproduce the biological processes investigated was demonstrated by their goodness of fit [14,17,30]. Although these equations were not validated against independent data, they were considered robust enough to be used in this model [83]. Because these previous papers showed that, the response of different strains belonging to the main transposon genotypes found in vineyards to environmental conditions was uniform, our model considered the *B*. *cinerea* population of a vineyard to be uniform.

The model describes three key stages of the *B*. *cinerea* life cycle in vineyards: i) production of conidia on overwintered sources; ii) infection of inflorescences and young clusters by conidia and production of conidia on bunch trash (pathway I and II of Elmer and Michailides [3]); and iii) infection of mature berries by conidia (pathway Va) and by mycelium (i.e., berry-to-berry infection; pathway Vb), as well as production of conidia on affected, ripening berries.

On a daily basis, the model predicts and then accumulates the relative infection severity over two infection windows corresponding to the two grape-growing periods relevant for *B*. *cinerea* infection: i) between “inflorescences clearly visible” and “berries groat-sized, bunches begin to hang” and ii) ripening berries [3].

The model was validated in 21combinations of vineyard × year in different grape-growing areas of Italy and in France. A DFA analysis was used to evaluate the ability of the model output (*SEV1*, *SEV2*, and *SEV3*) to predict whether Botrytis bunch rot epidemics were mild, intermediate, or severe. DFA has been previously used in botanical epidemiology to compare epidemics and to evaluate the importance of multiple influencing variables on epidemic development [84–86]. Classification of the epidemics into the three groups was based on a prior probability of group membership, which was the real proportion of the 21 epidemics that were mild, intermediate, or severe. Under this assumption, a vineyard has a lower probability to develop a mild *B*. *cinerea* epidemic than an intermediate or severe epidemic (i.e., 0.19 vs. 0.48 and 0.33, respectively). The prior probabilities found in the data set based on observations of the 21 epidemics were consistent with those of a larger data set resulting from the analysis of 111 papers published in the journal *Giornate Fitopatologiche* between 1962 and 2012 (http://fitogest.imagelinenetwork.com/it/giornate-fitopatologiche/). In this larger data set, 14% of the Botrytis bunch rot epidemics in untreated plots were mild, 59% were intermediate, and 26% were severe.

The DFA analysis correctly classified more than 80% of the 21 epidemics, indicating that the model output provided a reliable picture of reality. Three of the four misclassified epidemics were underestimated, i.e., the model predicted a lower level of disease than was observed in the field. Although the model predicts the development of Botrytis bunch rot epidemics based on grape phenology and environmental conditions, the severity of Botrytis bunch rot is influenced by other factors that were not included in the model. These other factors include wounds caused by abiotic factors [17,19,22,23,35] and by the grape moth *Lobesia botrana* [19,87,88], thrips [89], birds, and powdery mildew (*Erysiphe necator*) [35]. The severity of Botrytis bunch rot may also be influenced by cultural practices such as excessive nitrogen fertilization or irrigation [22]. These factors may have contributed to the model underestimation.

The results of the DFA showed that the variable *SEV1* had the most influence in determining the severity of *B*. *cinerea* rot on mature bunches. *SEV1* concerns *B*. *cinerea* infections occurring between “inflorescences clearly visible” and “berries groat-sized, bunches begin to hang”. These early season infections may result in inflorescence and young berry rot, in latent infections, and in bunch trash colonisation [3]. We assessed bunch rot at bunch maturity but did not evaluate the accuracy of *SEV1* in predicting rot of inflorescences and young clusters. Therefore, the relevance of *SEV1* observed in this work may be caused by an indirect estimation of: i) latent infections of young berries that may have occurred at flowering; ii) the amount of bunch trash represented by aborted, infected flowers and young berries; and iii) the incidence of bunch trash colonized by *B*. *cinerea*. These three effects all increase Botrytis bunch rot at harvesting. That latent infections contribute to final disease incidence has been established [25,55,57,60,61], but the extent to which the onset of latent infections contributed to final disease was inconsistent and ranged from 5 to 100%; this inconsistency is probably explained by differences in locations, grape varieties, and the methods used to assess latency.

DFA also showed that *SEV2* and *SEV3*, which account for the infections of ripening berries, had a minor effect on determining the severity of Botrytis epidemic. *SEV2* and *SEV3* relate to pathways Va and Vb (both were considered as pathway V by Elmer and Michaillides [3]), which have been previously considered to be relevant in several wine-growing regions [17,18,47,60,62,63,90,91]. *SEV2* estimates the risk for infection of ripening berries by conidia, whereas *SEV3* estimates the risk for berry-to-berry infection by aerial mycelium. The presence of colonized bunch trash and rotted berries within the bunch (sources of aerial conidia and mycelium) are essential for infection pathways Va and Vb, respectively. Thus, a high value of *SEV2* and/or *SEV3* (indicating favourable conditions for conidial and berry-to-berry infection, respectively) may result in no or low disease if there are no or few affected berries within the bunch. Because the presence of colonised bunch trash and the initial presence of infected berries during ripening are both indirectly accounted for by *SEV1*, the effect of *SEV2* and *SEV3* on bunch rot may also depend on *SEV1*. For this reason, an in-vineyard assessment of trash colonisation by *B*. *cinerea* during pre-bunch closure and/or of Botrytis rot incidence at veraison could greatly improve the accuracy of the model in predicting the infection severity of ripening berries.

DFA can also be used to predict the final bunch rot severity in an ongoing epidemic. Among the 21 epidemics considered in this work, prediction of the final disease severity changed from mild to intermediate before the end of flowering, and changed from intermediate to severe after veraison. Thus, it seems that *SEV1* was the most important variable for discriminating between mild and intermediate epidemics, whereas *SEV2* and *SEV3* determined the change between intermediate and severe epidemics.

Currently, fungicide treatments are mainly applied based on vine phenology, with four vine growth stages being considered critical: end of flowering (A), pre-bunch closure (B), veraison (C), and before harvest (D) [33,92,93]. This method is easy to follow and provides excellent protection against Botrytis bunch rot in different grape-growing areas [42, 92–98]. Unfortunately, these treatments do not take into account the real risk of bunch infection at each growth stage, and they lead to expensive, unnecessary fungicide sprays [42,96]. To identify high-risk periods for *B*. *cinerea* infection, researchers have proposed the use of empirical rules [32,33], weather-driven models [15,20,47], and an expert system [43]. However, few or no validation data exist for some of the weather-driven models [15,20,47], validation results were inconsistent for others [34,44–46], and no clear benefits have been demonstrated by the use of these methods [49].

The model proposed here: i) considers the key aspects of the *B*. *cinerea* life cycle; ii) integrates recent knowledge on host susceptibility at different growth stages and on the influence of weather on sporulation and on the main infection pathways; iii) is based on experiments [14,17,30] that considered the genetic variability of the *B*. *cinerea* populations in vineyards [12,99]; and iv) has been validated in different locations and under different environmental conditions. The model can then be regarded as an improvement of the previous Botrytis models in viticulture. Utility of the model in scheduling fungicides to control Botrytis bunch rot, however, should be verified with experiments [100–102]. After that, this new model could be used instead of the 15–15 rule or the previous models for making decisions about fungicide application at specific vine growth stages.

In the future, the model could be incorporated into existing decision support systems for sustainable vineyard management [38,103,104]. The model could also be improved with site-specific information affecting the susceptibility to *B*. *cinerea* infection. This site-specific information could include the susceptibility of specific grape varieties [48,73,105] and the factors that predispose berries to infection, such as wounds, insect attacks, incidence of other diseases, or cultural practices [17,19,22,23,35,89].

## Supporting Information

S1 Appendix Development of *SUS1*, *SUS2* and *SUS3* equations. (DOCX)
