# Supplementary material for: A Mechanistic Model of Botrytis cinerea on Grapevines That Includes Weather, Vine Growth Stage, and the Main Infection Pathways
Source: PLoS One. 2015 Oct 12;10(10):e0140444. doi: 10.1371/journal.pone.0140444 (PMC4601735; doi:10.1371/journal.pone.0140444)
Supplement: S1 Appendix — (DOCX) [file pone.0140444.s001.docx]

This Appendix describes the development of three equations that model the changes in host susceptibility to *B. cinerea* in the different infections pathways described in the model (i.e., *SUS1*, *SUS2* and *SUS3*). The equation parameters were estimated using the nonlinear regression procedure of SPSS (ver. 21.0, IBM SPSS Statistics, IBM Corp., New York, USA).

*SUS1* was developed based on the information contained in Ciliberti *et al*. [14], in which the effect of temperature and wetness duration on infection of grape inflorescences and young berry clusters by *Botrytis cinerea* was investigated. For that, inflorescences and young berry clusters were collected at seven growth stages: inflorescence clearly visible (stage 53 of the scale of Lorenz *et al*.[31]), inflorescence fully developed (stage 57), beginning of flowering (stage 61), full flowering (stage 65), end of flowering (stage 69), fruit set (stage 71) and berries groat-sized (stage 73); inoculations were performed by immersion in a conidial suspension of *B. cinerea*. The different samples were incubated at 20°C in darkness for 0, 3, 6, 12, 24, or 48 h. After that, the pieces were disinfested, dried, and incubated for 2 weeks. The area under the infection progress curve (AUIPC) was calculated, being incidence the proportion of inflorescence or cluster pieces showing *B. cinerea* sporulation. The experiment resulted in higher values of AUIPC for inflorescences inoculated during flowering (stages 61, 65, and 69). For each stage the values of AUIPC were 1.90 for stage 53, 2.19 for stage 57, 3.06 for stage 61, 4.00 for stage 65, 3.24 for stage 69, 2.65 for stage 71, and 2.37 for stage 73. These results were fitted to a polynomial by using a regression analysis, in the form: *y* = *a ×* *(GS/100)^3^* + *b ×* *(GS/100)^2^* + *c ×* (*GS/100)* +*d*; where: *y* = relative AUIPC referred to the maximum; *a, b, c*, and *d* = equation parameters; and *GS* = growth stage of the plant based on scale of Lorenz *et al.*[31]. The parameter estimates were *a*=-379.09, *b*=671.25, *c*=-390.33 and *d*=75.209; the model fitted the data with *R^2^* = 0.875 (Figure 7).

*SUS2* was developed based on the information contained in Deytieux-Belleau *et al*. [18], relative to the evolution of berry susceptibility during maturation. Briefly, these authors inoculated berries at four stages from veraison to harvest: mid-color change (stage 83), end of color change (stage 85), during maturation (stage 87.5) and full maturity (stage 89). Inoculations were performed by placing mycelial plugs of *B. cinerea* on the skin surface and incubated them at 15°C and 100% relative humidity (RH). Finally, the authors assessed the mean rot severity at 24 days post-inoculation (dpi), as the mean percentage of the berry surface expressing rot symptoms. The results of this experiment showed MRS of 8% at growth stage 83, 16% at stage 85, 38% at stage 87.5 and 93% at stage 89. These results were fitted to an exponential equation in the form: *y* = *a × e* ^(^*^b^* *^× GS^*^)^; where: y = MRS referred to the maximum MRS observed during the experiment; *a* and *b* = equation parameters; and *GS* = growth stage of Lorenz *et al*. [31]. The parameter estimates were *a*= 5 *x* 10^-17^and *b*= 0.4219; the equation fitted the data with *R^2^* = 0.976 (Figure 7), and the SE of the parameters were 0.051 for *a* and <0.0001 for *b*.

*SUS3* was developed based on one experiment from Ciliberti *et al*. [14]. The mycelial growth of *B. cinerea* strains was evaluated under different berry juice medium compositions similar to the following berry growth stages: pea-sized berries (stage 75), veraison (stage 83), softening of berries (stage 85), and ripe berries (stage 89) (Lorenz *et al*., [31]). Mycelium plugs were placed in the different media and incubated at 20°C for 20 days. Finally, the colony area was determined by measuring two perpendicular diameters. The average growth (cm) of the strains was: 1.48 for the medium similar to berries at stage 75, 4.19 for stage 83, 4.90 for stage 85, and 6.45 cm for stage 89. The linear regression between these two datasets was calculated in the form: *y*=*a* + *b* *×* *GS*, where: y = relative growth referred to the maximum growth observed in the experiment; *a* and *b* = equations parameters; and *GS* = growth stage of Lorenz *et al*. [31]. The parameter estimates were *a*= -3.87 and *b*= 0.05; the equation fitted the data with *R^2^* = 0.998 (Figure 7) and the SE of the parameters were 0.113 for *a* and 0.001 for *b*.

**Figure 7.** Predicted versus observed data on relative SUS1 (blue), SUS2 (red) and SUS3 (green). Data are predicted by the equations described above.
